# Combined Metabarcoding and Co-occurrence Network Analysis to Profile the Bacterial, Fungal and *Fusarium* Communities and Their Interactions in Maize Stalks

**DOI:** 10.3389/fmicb.2019.00261

**Published:** 2019-02-18

**Authors:** José Francisco Cobo-Díaz, Riccardo Baroncelli, Gaétan Le Floch, Adeline Picot

**Affiliations:** ^1^Laboratoire Universitaire de Biodiversité et Ecologie Microbienne, IBSAM, ESIAB, Université de Bretagne Occidentale, Plouzané, France; ^2^Instituto Hispano-Luso de Investigaciones Agrarias (CIALE), University of Salamanca, Salamanca, Spain

**Keywords:** maize residues, bacterial communities, fungal communities, *Fusarium* communities, biocontrol agents, co-occurrence network

## Abstract

Fusarium Head Blight (FHB) is one of the most devastating diseases of cereals worldwide, threatening both crop production by affecting cereal grain development, and human and animal health by contaminating grains with mycotoxins. Despite that maize residues constitute the primary source of inoculum for *Fusarium* pathogenic species, the structure and diversity of *Fusarium* spp. and microbial communities in maize residues have received much less attention than in grains. In this study, a metabarcoding approach was used to study the bacterial, fungal and *Fusarium* communities encountered in maize stalks collected from 8 fields in Brittany, France, after maize harvest during fall 2015. Some predominant genera found in maize residues were cereal or maize pathogens, such as the fungal *Fusarium*, *Acremonium*, and *Phoma* genera, and the bacterial *Pseudomonas* and *Erwinia* genera. Furthermore, a high predominance of genera with previously reported biocontrol activity was found, including the bacterial *Sphingomonas*, *Pedobacter*, *Flavobacterium, Pseudomonas*, and *Janthinobacterium* genera; and the fungal *Epicoccum*, *Articulospora*, *Exophiala*, and *Sarocladium* genera. Among *Fusarium* spp., *F. graminearum* and *F. avenaceum* were dominant. We also found that the maize cultivar and previous crop could influence the structure of microbial communities. Using SparCC co-occurrence network analysis, significant negative correlations were obtained between *Fusarium* spp. responsible for FHB (including *F. graminearum* and *F. avenaceum*) and bacterial OTUs classified as *Sphingomonas* and fungal OTUs classified as *Sarocladium* and *Epicoccum*. Considering that isolates belonging to these taxa have already been associated with antagonist effect against different *Fusarium* spp. and/or other pathogenic microorganisms and due to their predominance and negative associations with *Fusarium* spp., they may be good candidates as biocontrol agents. Combining the use of *Fusarium*-specific primers with universal primers for bacteria and fungi allowed us to study the microbial communities, but also to track correlations between *Fusarium* spp. and other bacterial and fungal genera, using co-occurrence network analysis. Such approach could be a useful tool as part of a screening strategy for novel antagonist candidates against toxigenic *Fusarium* spp., allowing the selection of taxa of interest.

## Introduction

Fusarium Head Blight (FHB) of cereals (Nazari et al., 2014) is caused by several *Fusarium* species among which *F. graminearum*, *F. culmorum*, *F. avenaceum*, and *F. poae* are the main causal agents in Europe (Xu et al., 2005; Hellin et al., 2016). FHB is one of the most important diseases affecting cereals worldwide (Ramirez et al., 2006; Bateman et al., 2007; Gong et al., 2015) and represents a threat to human and animal health due to the possible production of mycotoxins by *Fusarium* species (Desjardins and Proctor, 2007). Crop rotation, and in particular maize as previous crop, can increase the risk of FHB incidence as previous crop infected residues are the primary source of pathogenic species (Shaner, 2003; Bateman et al., 2007; Fernandez et al., 2008). A high incidence of *Fusarium* species was found in the first internode-stalk of maize plants (Scauflaire et al., 2011), which is usually left in the field, turning into a main inoculum source for the following crop (Maiorano et al., 2008). Current crop, cropping history and tillage system have a significant influence on *Fusarium* and fungal communities of crop residues (Fernandez et al., 2008), on maize rhizospheric microbiome (Benitez et al., 2017) and on bulk soil microbial communities (Legrand et al., 2018). Although the plant genotype affects the rhizosphere microbial communities in maize (Aira et al., 2010), no studies have focused on how maize genotype affect the phyllosphere or the crop residue microbiome.

The low efficacy of current control strategies, mainly based on agricultural practices including tillage and the use of less sensitive cultivars, is prompting the scientific community to seek alternatives. Among them, the application of biocontrol agent against *Fusarium* species has been one of the major focuses of current research due to their compliance with environmental standards. Several candidate antagonists have been developed after isolation of microbial strains from different parts of cereals, such as root rhizosphere from maize (Abiala et al., 2015) and barley (Abd El Daim et al., 2015), wheat anthers (Palazzini et al., 2007), seed endophytes from wheat (Díaz Herrera et al., 2016), endophytes from maize (Mousa et al., 2015), or even from maize residues (Luongo et al., 2005; Singh et al., 2009), agricultural soils (He et al., 2009), silages and forest soils (Baffoni et al., 2015). Generally, the isolation of antagonistic candidates is empirical and needs confrontation tests under laboratory conditions which are used to screen a high number of candidates before field evaluations (He et al., 2009; Schöneberg et al., 2015). The efficacy of antagonists is usually reduced under field conditions compared to laboratory conditions (Luongo et al., 2005; Crane et al., 2013; Schisler et al., 2015; Legrand et al., 2017), mainly because of the complex interactions of antagonists with their biotic and abiotic environment in the field. Alternatively, this stepwise approach may also result in the possible loss of isolates that does not pass laboratory selection step but have good efficacy under field conditions (Schöneberg et al., 2015). In the latter study, they found that *Clonostachys rosea*, a weak competitor in *in vitro* co-culture with two *F. graminearum* and one *F. crookwellense* strains, showed the best antagonist potential of the total 12 strains screened in the field and was the only one able to reduce FHB incidence when inoculated after the pathogen. To increase the efficacy, recent studies demonstrated a synergistic/antagonistic activity of cocktail strains, such as the use of seven species isolated from maize roots to increase the efficacy of protection against *F. verticillioides* in maize kernels (Niu et al., 2017); similar results have been reached using a consortium of individually non-antagonistic bacteria of *F. oxysporum* in *Arabidopsis thaliana* (Fujiwara et al., 2016). Such isolation approach, however, is time-consuming, and still lack of efficacy. Despite some promising results, only a limited number of FHB biocontrol agents are commercially available (Legrand et al., 2017).

These approaches could undoubtedly benefit from the use of -omics technologies to better describe the microbial community functioning and improve the screening of antagonist organisms. Indeed, we must first gain a deeper understanding of the microbiota to which pathogens are confronted, and study the diversity and structure of pathogens themselves, especially for complex pathosystems such as FHB. Such knowledge may help select more appropriate biocontrol strategies, adapted to the *Fusarium* and microbial communities, which may vary according to the pedo-climatic environment of the agroecosystem. Specific primers designed to track *Fusarium* communities in soils and in wheat kernels using Next Generation Sequencing (NGS) approaches have already been developed (Edel-Hermann et al., 2015; Karlsson et al., 2016) but no studies combined this approach with the use of universal primers for fungal and/or bacterial species. A few metabarcoding studies aimed at describing the microbial communities in maize crop bulk soils and/or rhizospheric soils (Peiffer et al., 2013; Li et al., 2014; Zhao et al., 2016), while other focused on the influence of the presence of maize residues on soil microbiota (Chen et al., 2015; De la Cruz-Barrón et al., 2017) but none were dedicated to the microbial communities found on maize crop residues. Yet, since the primary source of *Fusarium* spp. inoculum originates from infected maize crop residues, it is important to deepen the knowledge of microbial communities associated with maize stalks. Those communities may contribute to soil suppressiveness against *Fusarium* pathogenic species and may be a good source of potential antagonists. Such approaches have already been undertaken in suppressive soils to vanilla and banana Fusarium wilt disease (Fu et al., 2017; Xiong et al., 2017) and in suppressive maize stalks to Fusarium ear rot (Köhl et al., 2015).

In this context, the aims of the present study were to (i) describe the bacterial, fungal and *Fusarium* communities found in maize stalks collected from fields after harvest in Brittany, France, using a metabarcoding approach; (ii) determine whether agronomic factors including the cultivar and/or the previous crop influence the microbial community structure and diversity; (iii) correlate the abundance of *Fusarium* pathogenic species with other bacterial and fungal taxa, using SparCC co-occurrence network analysis, as a preliminary step to identify potential antagonist microorganisms.

## Materials and Methods

### Maize Stalk Sampling

**FIGURE 1:**
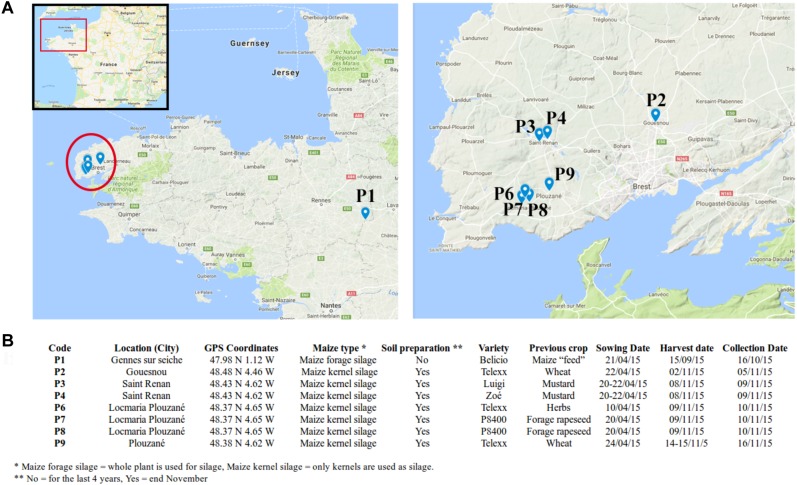
Field characteristics. **(A)** Location of the sampled fields; **(B)** agronomic characteristics of the fields.

Maize stalks were collected from eight agricultural fields across Brittany, France in November 2015. In total, seven fields were surveyed in Finistère (Gouesnou, Saint-Renan, Locmaria-Plouzané and Plouzané) and one in Ille-Et-Vilaine (Gennes sur Seiche) located approximately 300 km away from the other fields (Figure 1). Field characteristics including the maize types and varieties, the previous crop and tillage practices were recorded (Figure 1). In each field, the above-ground parts of 15 maize stalks with nodal region were randomly sampled. Stalks were sampled within 3 days after maize harvest and were stored at -80°C until DNA extraction, except the one in Ille-Et-Vilaine, which was sampled within a month after maize harvest. P1 was chosen as an outgroup to help interpret the degree of variability observed within the maize kernel silage fields from Finistère.

### DNA Extraction

For each field, 15 stalks were randomly chosen and 3 groups of 3 stalks were randomly selected at laboratory with each group corresponding to a biological replicate. For each replicate, four different portions of approximately 1 cm (nodal, internodal without leave, internodal with leave, and the external part) were cut with a sterilized scalpel from each stalk, mixed altogether and ground with liquid nitrogen in an autoclaved mortar and pestle. The pulverized tissues were stored in 1.5 mL Eppendorf tubes at 4°C until DNA extraction, performed within 4 h. DNA was extracted from 200 mg of pulverized maize stalks using FastDNA^®^SPIN kit (MP Biomedicals, Santa Ana, CA, United States) following the manufacturer’s instructions. Quality and concentration of purified DNA were determined using a UV spectrophotometer (NanoDrop 1000, Thermo Scientific, United States), and dilutions of at least 10 ng/μl were prepared for each DNA sample.

### PCR Amplification and MiSeq Sequencing

A total of 24 samples (8 fields × 3 replicates) were selected for amplicon PCRs and high-throughput sequencing. Preparation of 16S rRNA, ITS and TEF1 libraries, and Illumina MiSeq 300 PE sequencing were performed at the McGill University and Génome Québec Innovation Centre, Montreal, Canada. Primers 341F (5′-CCTACGGGNGGCWGCAG-3′) and 805R (5′-GACTACHVGGGTATCTAATCC-3′) (Herlemann et al., 2011) were used to amplify the variable regions V3 and V4 of the 16S rRNA gene; primers ITS1F (5′-CTTGGTCATTTAGAGGAAGTAA-3′) and ITS4 (5′-TCCTCCGCTTATTGATATGC-3′) (White et al., 1990; Gardes and Bruns, 1993) to amplify the internal transcribed spacer; and primers TEF_FUS_F6 (5′-CCGGTCACTTGATCTACCAG-3′) and TEF_FUS_R7 (5′-ATGACGGTGACATAGTAGCG-3′) (Cobo-Diaz, Baroncelli, Le Floch, Picot, unpublished) to amplify a 430 bp region of the translation elongation factor (TEF1) of *Fusarium* species.

### 16S rRNA Read Filtering

The raw sequences were processed and analyzed with QIIME v1.9.1 (Quantitative Insights Into Microbial Ecology) (Caporaso et al., 2010). After joining the paired-end reads using the *multiple_join_paired_ends.py* and *multiple_split_libraries_fastq.py* scripts with default parameters, the chimeric sequences were then removed using UCHIME algorithm (Edgar et al., 2011) implemented in VSEARCH v1.1.3^1^ against the ChimeraSlayer reference database (Haas et al., 2011). UCLUST algorithm (Edgar, 2010) was used for OTU picking and taxonomic assignment, which was made against GreenGenes v13.5 database (McDonald et al., 2012). To minimize the inflation of rare OTUs in the community analysis, we include only OTUs with sequence count greater than 10 (Brown et al., 2015; Oliver et al., 2015). Also, chloroplast, mitochondria and “No assigned” OTUs were discarded.

### ITS Read Filtering

Although expected, a low level of joined paired-end reads was obtained for the ITS dataset, leading us to choose a different approach using QIIME v1.9.1 (Caporaso et al., 2010). The forward and reverse files were merged independently, using *multiple_split_libraries_fastq.py*. ITS1 and ITS2 regions were first extracted separately from forward and reverse non-chimera-fasta files, respectively, using ITSx v1.0.11 (Bengtsson-Palme et al., 2013) before being concatenated in a new file. A chimera filtering was made on concatenated file using the UCHIME algorithm (Edgar et al., 2011) with VSEARCH v1.1.3 see text footnote^1^ and a modified version of the UNITE/INSDC representative/reference sequences version 7.2 (UNITE Community, 2017) as reference database. The modification consisted in extracting ITS1 and ITS2 regions by ITSx software and concatenated them in the modified version of the database.

The ITS1-ITS2 concatenated file of non-chimeric sequences was used for OTU picking running the QIIME script *pick_open_reference_otus.py*, with BLAST (Altschul et al., 1990) as taxonomic assignment method and a modified version of UNITE plus INSD non-redundant ITS database version 7.1 (Kõljalg et al., 2013). Again, the modified version consisted in concatenating ITS1 and ITS2 regions after extracting them using ITSx software.

To minimize the overestimation of rare OTUs in the community analysis, we include only OTUs with sequence count greater than 10 (Brown et al., 2015; Oliver et al., 2015). Only OTUs assigned to kingdom Fungi were used for further analysis. The taxonomy for fungi known to have both sexual and asexual stages was replaced by accepted names according to Chen et al. (2018).

### TEF1 Read Filtering

Paired-end reads were processed with QIIME (Caporaso et al., 2010), using the *multiple_join_paired_ends.py* and *multiple_split_libraries_fastq.py* scripts with default parameters. Pick *de novo* strategy was then employed to cluster the sequences into OTUs using *pick_de_novo_otus.py*, at 97% similarity cutoff. A first taxonomic assignment was performed using BLAST (Altschul et al., 1990) against NCBI non-redundant nucleotide database (nt)^2^. Only sequences assigned to *Fusarium* or the teleomorph name (*Gibberella* and *Nectria*), longer than 360 bp and with a percentage of identity higher than 97% were selected for further analysis, and only OTUs with sequence count greater than 10 were selected to minimize the inflation of rare OTUs in the community analysis (Brown et al., 2015; Oliver et al., 2015).

A second step of taxonomic assignment was done using the Fusarium MLST database web^3^, with the “pairse DNA alignments” tool, and compared with that provided by nt database. TEF1 sequences obtained along with references were aligned using MAFFT v7.304 (Katoh et al., 2017). Multiple sequence alignments were exported to MEGA7 (Kumar et al., 2016) and the best-fit substitution model was calculated for each separate sequence dataset. Using MrBayes 3.2.6 (Ronquist et al., 2012), the Markov chain Monte Carlo (MCMC) algorithm was performed to generate phylogenetic trees with Bayesian posterior probabilities for combined sequence datasets using the nucleotide substitution models determined by MEGA7 (Kimura 2-parameter with gamma distributed rate variation among sites [K2-G]). Four MCMC chains were run simultaneously for random trees for 2,000,000 generations (standard deviation of split frequencies between runs reached <0.01). Samples were taken every 500 generations. The first 25% of trees were discarded as burn-in phase of each analysis and posterior probabilities were determined from the remaining trees.

### Alpha and Beta-Diversity Analysis

Metabarcoding datasets obtained after filtering (V3-V4 region of 16S rRNA, ITS1-ITS2 concatenated regions and *Fusarium* TEF1 sequences) were processed equally. A single rarefaction, based on the sample with the lowest number of reads, was used for alpha-diversity analysis using *single_rarefaction.py* QIIME script. OTUs richness (observed_otus) and evenness (equitability or Pielou’s index) were calculated with *alpha_diversity.py* QIIME script. The statistical software R v2.9.10 was used to perform one-way ANOVA with Tukey HSD *post hoc* test, for statistical analysis. Differences with *p* < 0.05 were regarded as statistically significant.

Taxa relative abundances across samples were compared with STAMP (Statistical Analysis of Metagenomic Profiles) bioinformatics software v 2.1.3 (Parks et al., 2014), using the OTU table from QIIME pipeline without any rarefaction. Statistical significance of the differences between multiple group-samples were calculated using ANOVA test, Tukey-Kramer *post hoc* test at 0.95 confidence interval, and corresponding *p*-values were corrected by Benjamini-Hochberg FDR (Benjamini and Hochberg, 2000).

Canonical Correspondence Analysis (CCA) were made at OTU level using four different datasets: (i) grouping the replicates by field samples, (ii) removing sample P1, (iii) grouping only maize varieties Telexx and P8400, and (iv) grouping previous crop forage rapeseed, wheat and mustard; using R package Vegan. Analyses of variance (ANOVA) were made between fields, variety or previous crop.

Significant correlations between the relative abundance of bacterial, fungal and *Fusarium* OTUs were made using Sparse Correlations for Compositional data algorithm implemented in SparCC python module (Friedman and Alm, 2012) and corresponding networks were plotted using the R package qgraph (Epskamp et al., 2012). Only correlations with a R-corr absolute value greater than 0.3 and *p*-value less than 0.05 were plotted. For correlation analysis, the relative abundance per bacterial, fungal or *Fusarium* OTUs was calculated by dividing the number of sequences per OTU by the total number of amplicon sequences for each sample. Additionally, relative abundance of *Fusarium* OTUs obtained with TEF1 primers, was also divided by the percentage of ITS sequences assigned to *Fusarium* genus, in order to have an estimated relative abundance (percentage) of each *Fusarium* species (determined by TEF1) in the total fungal community (determined by ITS).

### Accession Numbers

All the raw reads have been deposited at the NCBI and are available under the Bioproject ID PRJNA394063^4^, with BioSample accession numbers from SAMN07348271 to SAMN07348278.

## Results

### Microbial Community Structure

**FIGURE 2:**
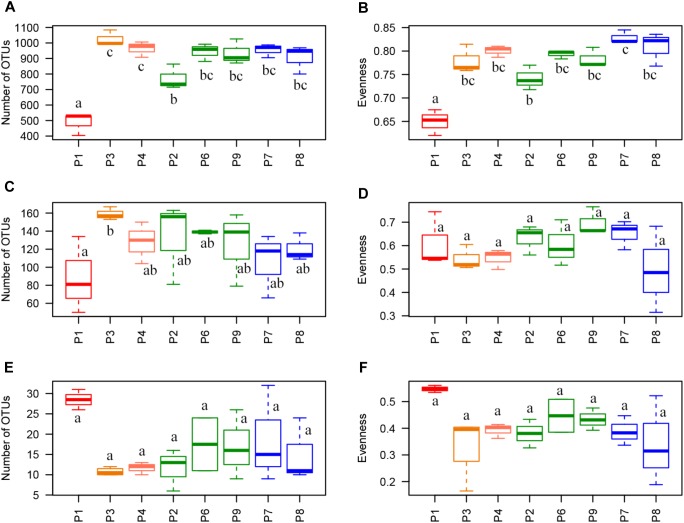
Alpha-diversity indices. Richness index (Observed OTUs) and evenness index (equitability or Pielou’s index) per sample for **(A,B)** 16S rRNA data, **(C,D)** ITS data, and **(E,F)** TEF1 data. Letters indicate statistical differences between samples (*p* < 0.05). Telexx variety samples are plotted in green and P8600 variety samples are plotted in blue.

A total of 1,041,456 sequences of 16S rRNA gene were clustered into 2,334 OTUs after filtering raw reads from 24 maize residue samples (8 fields × 3 biological replicates) and 12,936 sequences per sample were randomly extracted for alpha-diversity analysis. Only 1.98 and 4.54 % of the sequences were assigned to mitochondria and chloroplast, respectively, and removed along filtering step. Richness and evenness indices were significantly found the lowest in P1, with 488 observed OTUs, vs. 771 to 1,025 in the other samples, and with evenness value of 0.65 vs. 0.74 to 0.83. P2 also had significantly lower values compared to P3 and P4 for richness (771 OTUs vs. 964 OTUs in P4 and 1,025 in P3) and compared to P7 for evenness (0.74 vs. 0.83 in P7) (Figure 2A,B).

For ITS, 1,129,203 sequences were clustered into 455 OTUs and 29,319 sequences per sample were randomly extracted for alpha-diversity analysis. No sequences belonging to plants were detected. There were no significant differences for alpha-diversity indices between fields, with average values of richness between 88 and 159 OTUs, and 0.49 to 0.70 for equitability, except for the significantly higher values of observed richness found in P3 compared to P1 (159 vs. 88 OTUs, respectively) (Figure 2C,D).

In the case of TEF1 sequences assigned to *Fusarium*, a total of 1,023,229 sequences were clustered into 48 OTUs after raw read filtering. The percentage of sequences not assigned to the *Fusarium* genus was very low and only represented 0.039 % of the total sequences after removing those corresponding to phage phiX174, used for quality controls in Illumina sequencing. Between 33,688 and 56,829 sequences per sample (except replicate 1 from P1, which was removed for further analysis because it only had 15 sequences) were obtained, and 33,693 sequences per sample were randomly extracted for alpha-diversity analysis. Although P1 showed a higher value of evenness (0.55) and richness (28 OTUs) than other fields (evenness from 0.34 to 0.43 and richness from 11 to 19 OTUs), these differences were not statistically significant (Figure 2E,F).

### Bacterial Community Composition

**FIGURE 3:**
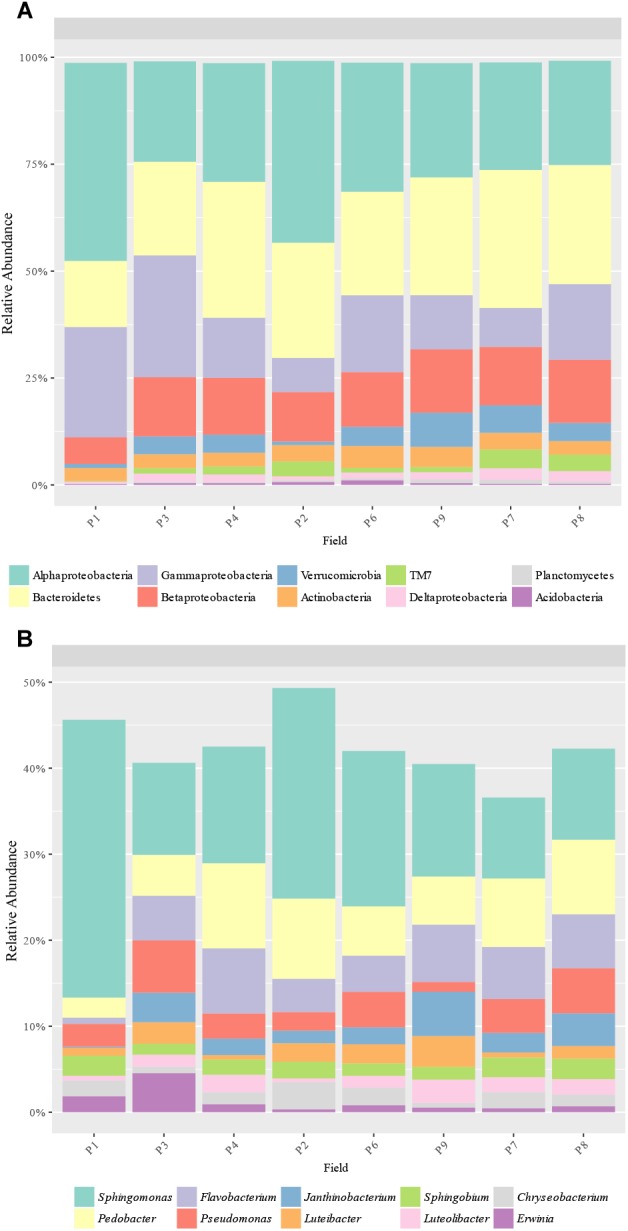
Bacterial taxa distribution. Relative abundance of the predominant **(A)** bacterial phyla (and Proteobacteria classes) and **(B)** genera obtained by 16S rRNA amplicons.

A total of 17 phyla and 143 genera were detected based on 16S rRNA sequences. The most abundant phyla were Proteobacteria (50.5–80.6%), Bacteroidetes (13.6–31.9%), Verrucomicrobia (0.8–8.0%), Actinobacteria (2.7–5.1%), and TM7 (0.2–4.4%), with Alphaproteobacteria (23.4–42.8%), Gammaproteobacteria (7.7–32.2%), and Betaproteobacteria (6.6–14.8%) as the main proteobacteria classes (Figure 3A). P1 had a significantly higher abundance of Proteobacteria (80.6% vs. 50.5 to 67.7%) and a lower abundance of Betaproteobacteria (6.6% vs. 11.4 to 31.9%), Bacteroidetes (13.6% vs. 21.9 to 31.9%), and Verrucomicrobia (1.3 and 0.8, vs. 4.1 to 5.1%) compared to the other fields. Alpha and Gamma-proteobacteria were also found to be higher in P1 although differences were not significant. Other significant differences were found in P3, where a higher abundance of Proteobacteria (67.7% vs. 50.5 to 62.8%) and Gammaproteobacteria (28.8% vs. 7.7 to 18.3%) and a lower abundance of Bacteroidetes (21.9% vs. 24.1 to 31.9%) were observed compared to the other samples, except P1 (Figure 3A).

**FIGURE 4:**
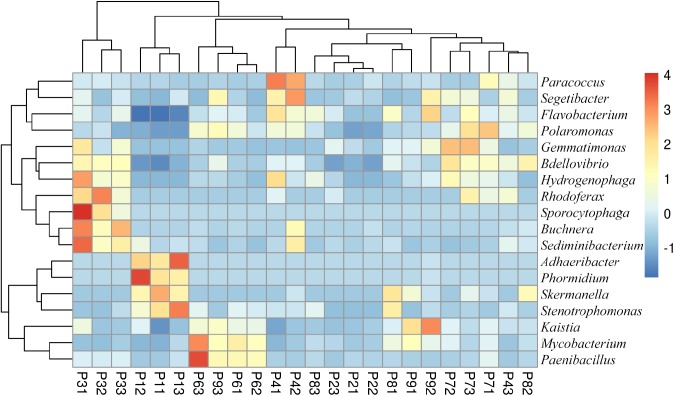
Heatmap of bacterial genera with significant differences (*p* < 0.05) between samples. Relative abundance data were z-scored normalized by row. Plot was made using *pheatmap* R package with the default parameters. Sample names have the format Pij, where i refers to the field site (*i* = 1–8) and j refers to the replicate within the field (*j* = 1–3).

The most abundant genera were 4 proteobacteria: *Sphingomonas* (9.6–27.9%), *Pseudomonas* (1.1–6.2%), *Janthinobacterium* (0.1–5.2%) and *Sphingobium* (1.3–2.4%); and 2 bacteroidetes: *Pedobacter* (2.1–9.8%) and *Flavobacterium* (0.6–7.7%) (Figure 3B). In total, the abundance of 18 genera was found to be significantly different between samples, with some genera more abundant in P1, such as *Adhaeribacter*, *Phormidium*, *Stenotrophomonas*, and *Skermanella* (Figure 4). Some genera were also found to be significantly more abundant in field P3 (*Rodhoferax*, *Sporocytophaga*, *Buchnera*, and *Sediminibacterium*), P4 (*Segetibacter* and *Paracoccus*), P6 (*Paenibacillus* and *Mycobacterium*), P7 (*Polaromonas*, *Bdellovibrio*, and *Gemmatimonas*), and P9 (*Kaistia*) (Figure 4).

### Fungal Community Composition

**FIGURE 5:**
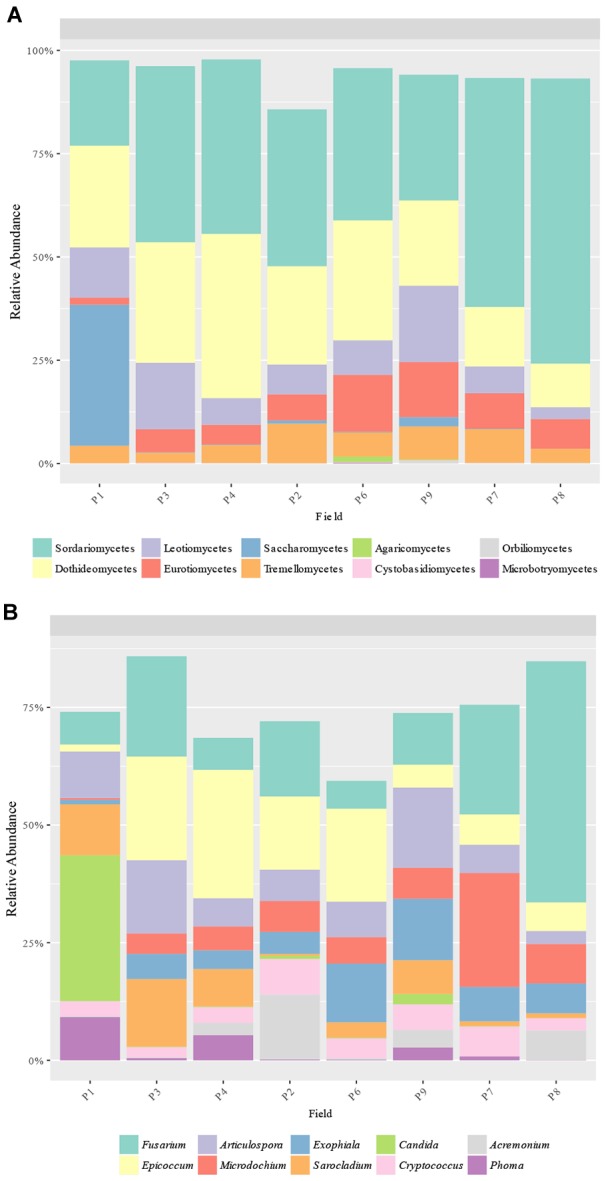
Fungal taxa distribution. Relative abundance of the predominant fungal **(A)** classes and **(B)** genera obtained by ITS amplicons.

A total of 16 classes and 124 genera were detected from ITS sequences. The most abundant classes were Sordariomycetes (20.3–63.1%), Dothideomycetes (12.5–39.6%), Leotiomycetes (3.6–18.0%), Eurotiomycetes (1.8–13.8%), Tremellomycetes (2.4–9.9%), and Saccharomycetes (0.0–33.0%), with Sordariomycetes being the most abundant class in all samples except in P1, which had Saccharomycetes as the main class (Figure 5A). In addition, Saccharomycetes was the unique class with different relative abundances between samples, which was more abundant in P1 than the others (33.0% vs. up to 2.3%). The most abundant genera obtained were *Fusarium* (6.1–44.8%), *Epicoccum* (1.4–27.1%), *Articulospora* (3.5–16.6%), *Microdochium* (0.3–24.6%), *Exophiala* (1.0–12.9%), *Sarocladium* (0.4–14.5%), *Cryptococcus* (2.1–7.7%), *Candida* (0.0–30.0%), *Acremonium* (0.1–11.5%), and *Phoma* (0.0–9.2%) (Figure 5B). Only 2 genera had significant different abundances between fields: *Candida* (class Saccharomycetes) and *Phoma* (class Dothideomycetes) were more abundant in P1 than other fields (30.0% vs. up to 2.4% and 9.2 vs. up to 5.4%, respectively) (Figure 5B).

### Fusarium Community Composition

**FIGURE 6:**
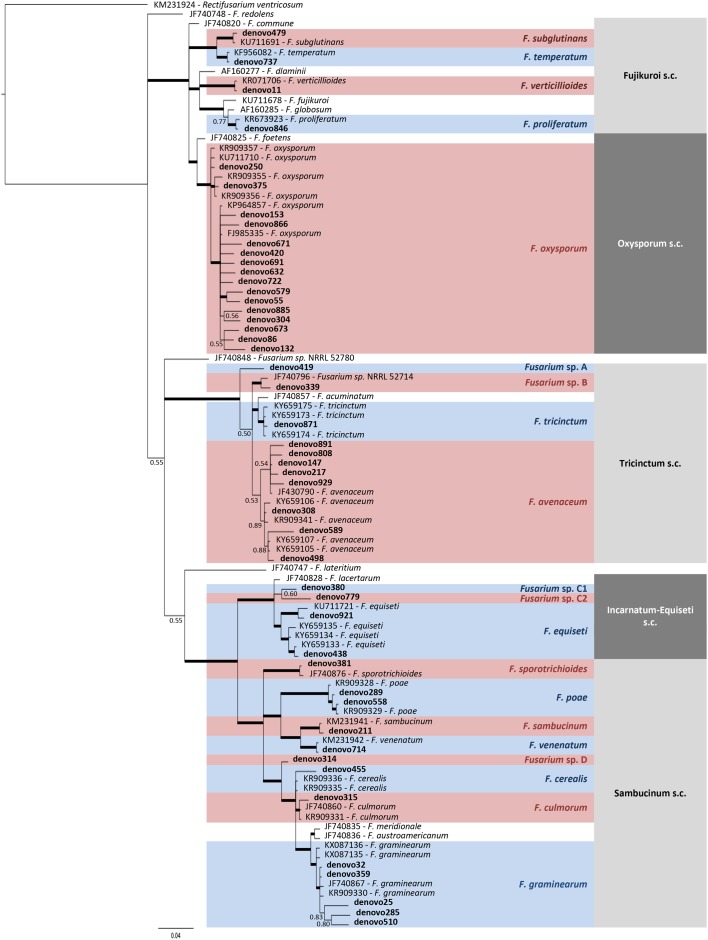
Phylogenetic tree of TEF1 sequences from *Fusarium* OTUs and reference isolates, which accession number was indicated. Bayesian posterior probability (BPP) values (above 0.50) are shown at the nodes. The thickened nodes represent BPP values higher than 0.9.

A total of 15 *Fusarium* species were detected according to taxonomic assignment performed by phylogenetic tree, although 5 OTUs were not clearly assigned to species level (Figure 6 and Supplementary Table S1). Three of them (denovo419, denovo339, and denovo314) were named as *Fusarium* sp. for further analysis, while denovo380 and denovo779 were grouped with the 2 OTUs assigned to *F. equiseti* (denovo921 and denovo438) inside *Fusarium* sp. FIESC (*Fusarium incarnatum-equiseti* species complex) for further analysis.

**FIGURE 7:**
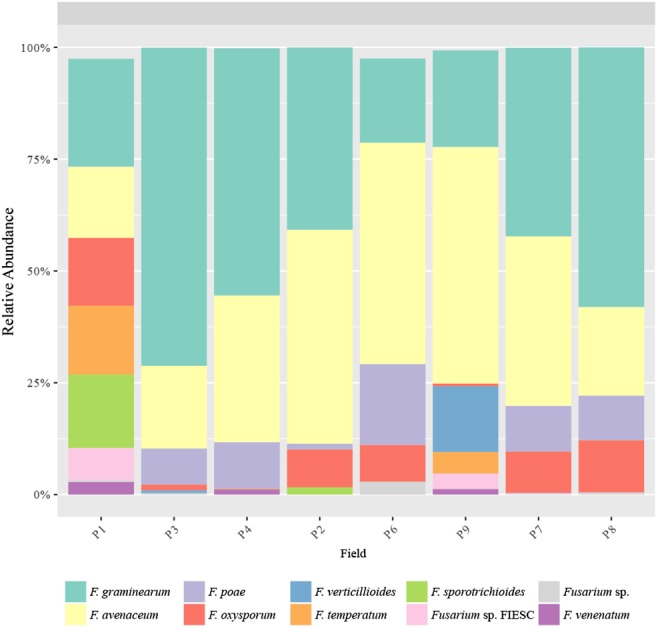
*Fusarium* species distribution. Relative abundance of the predominant *Fusarium* species obtained by TEF1 amplicons.

Major species were *F. graminearum* (18.9–71.9%), *F. avenaceum* (15.7–53.8%), *F. poae* (0.0–18.6%), *F. oxysporum* (0.002–12.5%), *F. verticillioides* (0.0–13.4%), *F. temperatum* (0–10.1%), and *F. sporotrichioides* (0.0–11.1%), which covered between 80.2 to 100% of the total *Fusarium* sequences per field (Figure 7). The abundance of these species was unevenly distributed across fields. However, whatever the field, the most abundant species were *F. graminearum* and *F. avenaceum*, which sum accounted for 68.9 to 90.1% of the total *Fusarium* sequences per field, except in P1 (46.6%), and they were also the 2 *Fusarium* species present in all the fields. It is noteworthy that *F. avenaceum* outnumbered *F. graminearum* only in the three fields that had Telexx as maize variety (P2, P6, and P9). Moreover, other species were also subdominant depending on the field: *F. poae* in P4, P6, P7, and P8 (from 9.2 to 18.6%); *F. oxysporum* in P1 and P8 (15.4 and 11.8%, respectively); *F. sporotrichioides* and *F. temperatum* in P1 (16.6 and 15.2%, respectively); and *F. verticillioides* in P9 (13.4%); although there were not significant differences in relative abundance of *Fusarium* species between samples (Figure 7).

### Canonical Correspondence Analysis

**FIGURE 8:**
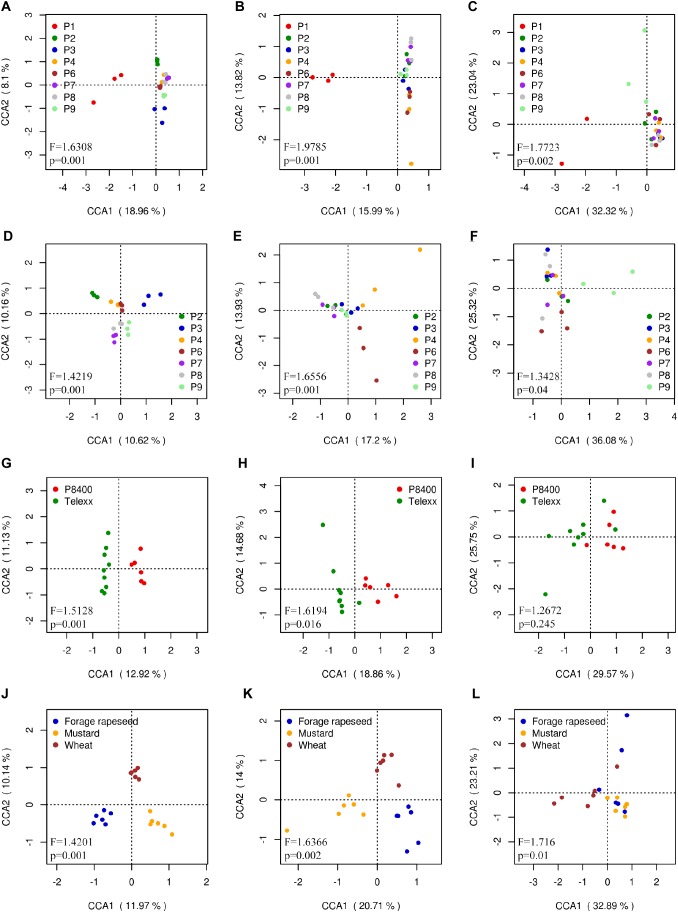
Canonical correspondence analysis (CCA) calculated using OTUs relative abundance. Each dot represents a sample replicate for **(A,D,G,J)** bacterial, **(B,E,H,K)** fungal, and **(C,F,I,L)** *Fusarium* communities, using **(A,B,C)** all sites, **(D,E,F)** all sites except P1, **(G,H,I)** samples grouped by maize variety and **(J,K,L)** by previous crop.

Unsurprisingly, Canonical Correspondence Analysis (CCA) showed that the composition and distribution of microbial communities in the outgroup P1 was different from the others samples. Bacterial, fungal and *Fusarium* communities presented significant differences between fields (ANOVA: *F* = 1.6308, Pr < 0.001 for bacteria; *F* = 2.0422, Pr < 0.001 for fungi; *F* = 1.7723, Pr < 0.001 for *Fusarium*), with P1 as the most different field in all 3 cases; and also P2, P3, and P9 for bacteria, and P9 for *Fusarium* communities (Figure 8A–C). The CCA excluding P1 showed similar degree of significance between samples (*F* = 1.4219 and 1.6738, for bacterial and fungal, respectively, Pr < 0.001), except for *Fusarium* (*F* = 1.3428, Pr = 0.038). A clear separation of almost all fields was observed for bacterial communities; P4 and P6 for fungal communities; and P6 and P9 for *Fusarium* communities (Figure 8D–F). The same analysis was done selecting the fields which had Telexx and P8400 as maize varieties, obtaining significant differences between varieties in bacterial (*F* = 1.5128, Pr = 0.002) and fungal (*F* = 1.7022, Pr = 0.015) communities but not in *Fusarium* communities (*F* = 1.2672, Pr = 0.214) (Figure 8G–I). Regarding the previous crop, only forage rapeseed, mustard and wheat were analyzed, resulting in significant differences between groups in bacterial (*F* = 1.4201, Pr = 0.001), fungal (*F* = 1.7673, Pr = 0.001) and *Fusarium* communities (*F* = 1.716, Pr = 0.01) (Figure 8J–L).

### SparCC Correlation Network

**FIGURE 9:**
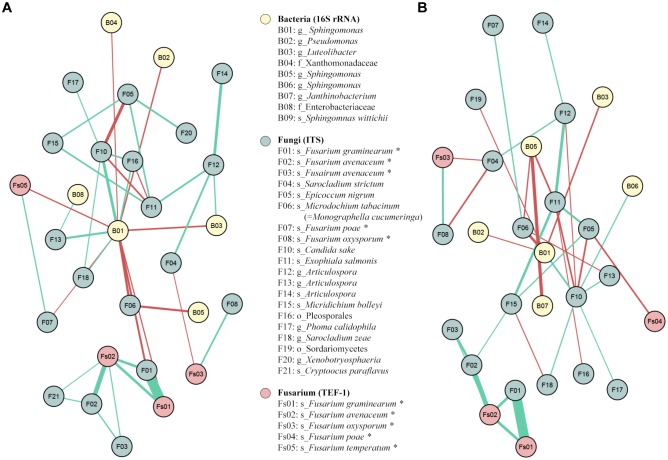
SparCC correlation networks observed between OTUs, obtained with 16S rRNA gene (Bacteria), ITS (Fungi), and TEF1 (*Fusarium*) sequences. Nodes correspond to OTUs, and connecting edges indicate correlations between them. Only nodes with negative or positive correlations, with values less than –0.30 (red) or larger than 0.30 (green), were represented. **(A)** SparCC correlation network using all the samples; **(B)** SparCC correlation network without the outgroup P1. OTUs marked with asterisk had been reassigned taxonomically using blastn against nr/nt-NCBI database and *Fusarium* MLST web database (Supplementary Table S2).

In total, 26 positive and 13 negative significant correlations (|coefficient correlation (=corr)|>0.3 and *p*-value < 0.05) were found between 27 OTUs: 6 from 16SrRNA, 19 from ITS and 4 from TEF1 (Figure 9A). The highest positive correlations were obviously found between F01 and Fs01, both assigned to *F. graminearum* by ITS and TEF1 sequencing, respectively (corr = 0.82); and between F02 and Fs02, both assigned to *F. avenaceum* by ITS and TEF1 sequencing (corr = 0.51). There were also significant positive correlations between Fs02 and the 2 *F. graminearum* OTUs (F01 and Fs01, corr = 0.40 for both) (Figure 9A). One fungal and one bacteria OTUs presented negative correlations with some *Fusarium* OTU(s): F04, assigned to *Sarocladium strictum*, against Fs03, assigned to *F. oxysporum* (corr = -0.30); and B01, assigned to *Sphingomonas*, against F01, Fs01, and Fs04, assigned to *F. graminearum* by ITS and TEF1, and to *F. poae* by TEF1 (corr = -0.36, -032 and -0.32, respectively). Moreover, B01 also presented negative correlations with B02, B03, B04, F06, and F07, assigned to *Pseudomonas*, *Luteolibacter*, family Xanthomonadaceae, *Monographella cucumerina*, and *F. poae*, respectively (Figure 9A).

The same SparCC correlation analysis was done excluding the results from the outgroup field P1, which fungal and bacterial communities and distribution greatly varied from the other fields. In total 28 OTUs (7 from 16SrRNA, 18 from ITS and 3 from TEF1) presented 20 positive and 16 negative significant correlations between them (Figure 9B). As happened in the previous network analysis, the highest positive correlations were between F01 and Fs01 (corr = 0.83), assigned to *F. graminearum* by ITS and TEF1, respectively; between F02 and Fs02 (corr = 0.53), assigned to *F. avenaceum* by ITS and TEF, respectively; and between F02 and the two OTUs assigned to *F. graminearum* (corr = 0.41 for both TEF1 and ITS). Two fungal OTUs had negative correlations vs. some *Fusarium* OTU: the same *Sarocladium* OTU identified before (F04) vs. Fs03 and F08, assigned to *F. oxysporum* by TEF1 and ITS sequences (corr = -0.31 and -0.35, respectively); and F05, assigned to *Epicoccum nigrum*, vs. Fs04 and F10, assigned to *F. temperatum* and *Candida sake* (corr = -0.34 and -0.35, respectively) (Figure 9B).

## Discussion

By sequencing three different amplicons from 24 maize samples, a total of 2,334 bacterial OTUs, 1,428 fungal OTUs and 48 *Fusarium* OTUs were obtained. This study is one of the first metabarcoding studies on maize residues, resulting in a higher diversity than previously found in other NGS studies of the maize rhizosphere (Peiffer et al., 2013; Li et al., 2014).

Proteobacteria (mainly alpha and gamma-proteobacteria), Bacteroidetes and Actinobacteria were found as the most abundant phyla in maize stalk surface, the first two of which have already been reported as the most abundant in the maize rhizosphere (Peiffer et al., 2013; Li et al., 2014), in soil samples after maize harvesting (Chen et al., 2015) and in the first stages of maize straw decomposition (Sun et al., 2013). Although maize residues can be considered as a separate compartment and a particular ecological niche compared to soil samples, some of the more abundant bacterial genera in the present study had also been found as predominant in maize rhizospheric soils, including *Sphingobium* (Peiffer et al., 2013; Li et al., 2014), *Flavobacterium* (Li et al., 2014; Yang et al., 2017; Correa-Galeote et al., 2016) and *Sphingomonas* (Correa-Galeote et al., 2018). Apart from these genera, the diversity of bacterial communities found in our maize residues generally differed from that of maize rhizospheric soils found in other studies (Peiffer et al., 2013; Li et al., 2014; Correa-Galeote et al., 2016; Yang et al., 2017). It should also be underlined that 2 of the over-represented genera in P1, *Adhaeribacter* and *Stenotrophomonas*, were also significantly more abundant in Fusarium wilt suppressive soils (Siegel-Hertz et al., 2018).

Some of the most abundant fungal genera found in the present study, including *Fusarium*, *Epicoccum*, *Acremonium*, *Sarocladium*, and *Phoma* had been reported as endophytes isolated from maize (Pan and May, 2009; Amin, 2013; Xing et al., 2018). In particular, *Fusarium*, *Epicoccum*, and *Phoma* were reported as the most abundant endophytes isolated from maize leaves, using two different lineages (Szilagyi-Zecchin et al., 2016), while *Acremonium* was detected exclusively in Fusarium wilt suppressive soils, but not in conducive soils (Siegel-Hertz et al., 2018). It is important to highlight that, in our maize samples, genera with reported biocontrol activity (Table 1) were predominant and included several bacterial genera such as *Sphingomonas*, *Pedobacter*, *Flavobacterium*, *Pseudomonas*, *Janthinobacterium*, *Sphingobium*, *Chryseobacterium*, *Luteibacter*, *Dyadobacter*, and *Rhizobium;* and fungal genera such as *Epicoccum*, *Articulospora*, *Exophiala*, *Sarocladium*, *Cryptococcus*, *Candida*, *Acremonium*, and *Metschnikowia*. On the other hand, only two bacterial genera, *Pseudomonas* and *Erwinia*, and three fungal genera, *Fusarium*, *Acremonium*, and *Phoma*, within the most abundant ones are known to include maize or cereal pathogens. *A priori*, although maize residues have been reported as the primary source of pathogenic species (Shaner, 2003; Bateman et al., 2007; Fernandez et al., 2008), microbial communities obtained from maize crop residues presented an important amount of organisms that could increase the protection of future rhizospheric complexes against pathogens. This hypothesis was corroborated with the presence of one bacterial and two fungal OTUs, assigned to *Sphingomonas*, *Epicoccum nigrum*, and *Sarocladium strictum*, negatively correlated to some *Fusarium* OTUs; these three genera were within the most abundant ones in the microbial communities studied. Likewise, a lower increase of *Fusarium* spp. colonization of maize stalks has also been reported when *Sphingomonas* species were more abundant (Köhl et al., 2015) and a strain of *Sphingomonas* was found to be antagonistic against *F. avenaceum*, *F. culmorum*, *F. tricinctum*, and *F. graminearum* (Wachowska et al., 2013). *Acremonium* spp. (basionym of *Sarocladium*) (Summerbell et al., 2011) were also found to be more abundant in maize stalks characterized by a lower increase in *Fusarium* colonization, rendering it as a potential antagonist of *Fusarium* spp. (Köhl et al., 2015). A better taxonomic classification of the *Acremonium* species present in our samples might be necessary as some species belonging to this genera have been reported to be pathogenic on maize (Tagne et al., 2002). Several *Acremonium* strains isolated from maize were able to inhibit the growth of some pathogens, such as *Pythium ultimum*, *Sclerotium oryzae*, *Rhizoctonia solani*, and *Pyricularia oryzae* (Potshangbam et al., 2017) or produce pyrrocidines A and B, which induce host defense mechanisms against microbial pathogens (Wicklow and Poling, 2009). Some *Sarocladium* endophytes isolated from wheat were also reported to inhibit *F. graminearum* and *F. culmorum* growth (Comby et al., 2017). It was also found that some strains of *Epicoccum nigrum* were able to reduce the mycelial growth of *F. graminearum*, *F. avenaceum*, and *F. oxysporum* on PDA (Ogórek and Plaskowska, 2011) and also in sterile wheat grain assays with *F. graminearum* (Jensen et al., 2016); or to reduce the sporulation of *F. culmorum* and *F. graminearum* on wheat straw (Luongo et al., 2005). Furthermore, *E. nigrum* has been used as a biological control on peaches and nectarines orchards against *Monilinia* spp. (De Cal et al., 2009) and against *Pythium debaryanum* and *P. ultimum* on cotton seedlings (Hashem and Ali, 2004). Overall, the high presence of these genera, previously reported as antagonists and negatively correlated to toxigenic *Fusarium* species or other pathogenic organisms, suggests that such taxa may be of interest as part of biocontrol strategies against toxigenic *Fusarium* spp.

**Table 1:** Pathogen and antagonistic characteristics of species or strains belonging to the most abundant genus obtained in maize residues.

	% of total	Plant	Wheat	Maize	Biocontrol
	sequences	pathogen	pathogen	pathogen	activity	Reference for biocontrol activity
**Bacterial genus**						
*Sphingomonas*	16.0	Yes	No	No	Yes	Wachowska et al., 2013
*Pedobacter*	6.7	No	No	No	Yes	De Boer et al., 2007; Song et al., 2017
*Flavobacterium*	5.1	No	No	No	Yes	Gunasinghe et al., 2004
*Pseudomonas*	3.6	Yes	Yes	Yes	Yes	Hennessy et al., 2017
*Janthinobacterium*	2.5	No	No	No	Yes	Berg et al., 2001
*Sphingobium*	1.9	Yes	No	No	Yes	van Bruggen et al., 2014; Fu et al., 2017
*Chryseobacterium*	1.7	Yes	No	No	Yes	Yin et al., 2013; Sang et al., 2018
*Luteibacter*	1.7	No	No	No	Yes	De Boer et al., 2007
*Luteolibacter*	1.5	No	No	No	No	
*Erwinia*	1.3	Yes	Yes	Yes	No	
*Agrobacterium*	1.3	Yes	No	No	(1)	
*Hymenobacter*	1.2	No	No	No	No	
*Dyadobacter*	1.0	No	No	No	Yes (2)	Fu et al., 2017
*Rhizobium*	0.8	No	No	No	Yes (3)	Al-Ani et al., 2012
**Fungal genus**						
*Fusarium*	17.1	Yes	Yes	Yes	Yes (3,4)	Ghini et al., 2000; Boari and Vurro, 2004
*Epicoccum*	13.1	Yes	No	No	Yes	Luongo et al., 2005
*Articulospora*	9.1	No	No	No	Yes	Sugahara et al., 2008
*Microdochium*	7.6	Yes	Yes	No	No	
*Exophiala*	7.0	No	No	No	Yes	Duvick et al., 1998
*Sarocladium*	5.7	Yes	No	No	Yes	Comby et al., 2017
*Cryptococcus*	4.4	Yes	No	No	Yes	Schisler et al., 2015
*Candida*	4.2	No	No	No	Yes	Calvo-Garrido et al., 2013
*Acremonium*	3.1	Yes	Yes	Yes	Yes (3)	Rajakumar et al., 2005
*Phoma*	2.4	Yes	Yes	Yes	No	
*Xenobotryosphaeria*	2.2	No	No	No	No	
*Pyrenochaetopsis*	2.2	No	No	No	No	
*Ramularia*	0.6	Yes	No	No	No	
*Hannaella*	0.5	Yes	No	No	No	
*Metschnikowia*	0.4	No	No	No	Yes	Manso and Nunes, 2011


*(1) Used for *Agrobacterium*-mediated plant transformation (Genetically Modified Organisms).*

*(2) Associated to disease suppressiveness.*

*(3) Over-represented in *Fusarium* wilt suppressive soils (Siegel-Hertz et al., 2018).*

*(4) Non-pathogenic strains.*

The most abundant *Fusarium* species found in our maize residues were *F. graminearum*, *F. avenaceum*, and *F. poae*, with 41.3, 35.4, and 7.1% of the total sequences, respectively, which are one of the main causal agents responsible for FHB (Parry et al., 1995; Bottalico and Perrone, 2002). Likewise, *F. graminearum* and *F. avenaceum* were also described as the predominant species on maize stalks after a 6-month exposure period in the field, as evaluated by qPCR (Köhl et al., 2015) and are commonly found as the main *Fusarium* species in wheat, using TEF1 as well as *Fusarium* spp. specific primers (Karlsson et al., 2016, 2017) or using culture-dependent approaches (Xu et al., 2005; Nicolaisen et al., 2014; Basler, 2016). *F. avenaceum* was also found as the dominant *Fusarium* species in some soil samples associated to perennial plants (LeBlanc et al., 2017). In opposite to our findings, *F. culmorum* is commonly described as a dominant *Fusarium* species in maize or cereals (Scauflaire et al., 2011; Basler, 2016; Hellin et al., 2016). Mutual exclusion between strains of *F. graminearum* and *F. culmorum* had been previously demonstrated (Siou et al., 2015). This competition could account for the low abundance of *F. culmorum* (0.05%) in maize stalks in our present study. In addition, shifts from *F. culmorum* to *F. graminearum* on wheat have been described in different European countries, such as England and Wales (Jennings et al., 2004), Netherlands (Waalwijk et al., 2003), Denmark (Nielsen et al., 2011), and Belgium for maize ears and stalks (Scauflaire et al., 2011). Climatic changes were the main hypothesis put forward, although the increase in maize-wheat rotation crops may also contribute to this increase in *F. graminearum* and decrease in *F. culmorum* (Dill-Macky and Jones, 2000; Cromey et al., 2002). The distribution of the *Fusarium* communities also strongly depends on the environmental conditions which would favor some species over the others. For instance, Dorn et al. (2011) found that *F. graminearum*, *F. verticillioides*, and *F. proliferatum* were the most dominant *Fusarium* species isolated from maize kernels in Switzerland (22.3–81.6, 2.5–41.8, and 0.6–22.0% of occurrence in kernels, respectively), while *F. equiseti*, *F. proliferatum*, and *F. verticillioides* were the main *Fusarium* species in stalks (43.6, 16.3, and 11.5% of occurrence in nodes, and 27.6, 41.5, and 15.5% in internodes, respectively). In our study, *F. graminearum* and *F. avenaceum* seem to be more adapted than species from the *Fusarium fujikuroi* species complex (FFSC), including *F. verticillioides* and *F. temperatum*, which were dominant in maize kernels in Poland (Czembor et al., 2015) and in maize kernels from Southern to Central Europe (Logrieco et al., 2002). The mild and humid climatic conditions found in Brittany, which are known to be favorable to *F. graminearum* and *F. avenaceum* (Xu et al., 2008) may account for such observations. In contrast, higher prevalence of Fusarium ear rot caused by FFSC including *F. verticillioides* occurs under hot and dry conditions. For instance, *F. verticillioides* incidence was found negatively correlated to rainfall values in maize fields in Argentina (Pereira et al., 2011) and to kernel moisture in maize fields in United States (Bush et al., 2004). Moreover, we found that Telexx maize variety had highest abundance of *F. avenaceum* than *F. graminearum*, while the opposite was found for the other varieties used in the study. These differences in *Fusarium* composition could also induce differences in mycotoxin concentrations, as deoxynivalenol and zearalenone are produced by *F. graminearum* while moniliformin, enniatins and beauvericin can be produced by *F. avenaceum* (Ferrigo et al., 2016).

Microbial communities found in the maize residues on field P1 was the most different compared to the other fields. In general, this field was characterized by both significant lower bacterial and a higher *Fusarium* alpha-diversity indices, with a higher abundance of the fungal *Candida*. Several factors differed from the outgroup P1 compared to the other fields (Table 1) including the maize type and cultivar, the agricultural practices, the previous crop and the location. In addition, these samples were collected 1 month after harvest, suggesting that maize residues were already in the process of degradation. Maize genotype has already been reported to influence the microbial communities in rhizospheric samples (Li et al., 2014), it could also have a strong influence in the microbial communities on others parts of the plant including the residues. But due to bias in the sampling design (because this study was rather designed to estimate the diversity found in maize residues from various fields in Brittany), we cannot conclude which factor(s) contributed mainly to these differences between P1 and the other fields. Additional sampling will be undertaken to further clarify which factor(s) has(have) the higher influence on microbial communities, with an emphasis to maize microbial dynamics over the course of maize residue degradation.

The significance of this study first lies in its design of a new specific pair of primers to identify *Fusarium* species with metabarcoding approach. This new culture-independent approach for *Fusarium* species identification could be adapted to other genera, by the design of specific primers for Illumina metabarcoding. In addition, the combined used of these primers with universal primers for fungi and bacteria allowed, not only to provide an accurate description of the microbiota as well as the pathogenic *Fusarium* spp. under various agronomic practices (maize cultivar, previous crop), but also to assess the potential relationships between microorganisms using co-occurrence network analysis. More particularly, we could identify predominant taxa negatively correlated to toxigenic *Fusarium* spp. Therefore, such approach could be used as a pre-filtering for the selection of potential antagonists as part of biocontrol strategies. Following this investigation, culture-dependent approaches must be done to determine the antagonistic potential of species identified by the co-occurrence network analysis, both in laboratory and field experiments. Illumina technology allows putting more than one amplicon type and dozens of samples in only one run (Herbold et al., 2015). This approach is time-saving compared to the empirical BCA isolation strategies, and could have more importance in the screening of antagonists.

Based on the results of this preliminary study, we also suggest focusing on the microbial dynamics throughout the plant cultivation cycle in maize-wheat rotations, taking also into account the influence of plant cultivar on microbial communities.

## Author Contributions

AP and GLF contributed to the conception and design of the study. AP performed sampling. JC-D performed DNA extraction and shipping, read filtering and OTU table filtering, and wrote the first draft of the manuscript. JC-D and GLF performed statistical analysis. RB performed phylogenetic tree. All authors contributed to manuscript revision, read and approved the submitted version.

## Conflict of Interest Statement

The authors declare that the research was conducted in the absence of any commercial or financial relationships that could be construed as a potential conflict of interest.

## Footnotes

1 https://github.com/torognes/vsearch

2 ftp://ftp.ncbi.nlm.nih.gov/../blast/db/

3 http://www.westerdijkinstitute.nl/fusarium/

4 http://www.ncbi.nlm.nih.gov/

## Supplementary Material

Table S1 Taxonomic assignment of representative TEF1 sequences for each *Fusarium* OTU obtained, using phylogenetic tree, blastn vs. nt-NCBI database, and pairse alignment vs. FUSARIUM-MLST database.

Table S2 Taxonomic assignment of representative TEF1 and ITS sequences for each *Fusarium* and unclassified genus OTUs plotted in network analysis, using blastn vs. nt-NCBI database, and pairse alignment vs. FUSARIUM-MLST database.
